# Progress in reducing child mortality and stunting in India: an application of the Lives Saved Tool

**DOI:** 10.1093/heapol/czz088

**Published:** 2019-09-16

**Authors:** Harold Alderman, Phuong Hong Nguyen, Purnima Menon

**Affiliations:** Division of Poverty, Health and Nutrition, International Food Policy Research Institute (IFPRI), 1201 I Street NW, Washington, DC, USA

**Keywords:** India, Lives Saved Tool, mortality, stunting, undernutrition

## Abstract

The Lives Saved Tool (LiST) has been used to estimate the impact of scaling up intervention coverage on undernutrition and mortality. Evidence for the model is largely based on efficacy trials, raising concerns of applicability to large-scale contexts. We modelled the impact of scaling up health programs in India between 2006 and 2016 and compared estimates to observed changes. Demographics, intervention coverage and nutritional status were obtained from National Family and Health Survey 2005–6 (NFHS-3) for the base year and NHFS-4 2015–16 for the endline. We used the LiST to estimate the impact of changes in coverage of interventions over this decade on child mortality and undernutrition at national and subnational levels and calculated the gap between estimated and observed changes in 2016. At the national level, the LiST estimates are close to the actual values of mortality for children <1 year and <5 years in 2016 (at 41 vs 42.6 and 50 vs 56.4, respectively, per 1000 live births). National estimates for stunting, wasting and anaemia at are also close to the actual values of NFHS-4. At the state level, actual changes were higher than the changes from the LiST projections for both mortality and stunting. The predicted changes using the LiST ranged from 33% to 92% of the actual change. The LiST provided national projections close to, albeit slightly below, actual performance over a decade. Reasons for poorer performance of state-specific projections are unknown; further refinements to the LiST for subnational use would improve the usefulness of the tool.

**Key Messages**

- Various studies have used the Lives Saved Tool (LiST) to project the impact of bringing key health interventions to scale. Given expansion of programs for which meta-analyses have been undertaken the tool provides estimates of lives saved as well as changes in nutritional indicators.
- Despite the fact that the parameters of the LiST come from comparatively modest scaled trials, the tool provides plausibly accurate aggregate projections of the impact of service expansion compared with actual trends over periods as long as a decade.
- The tool, however, may be less reliable at subnational levels, as indicates with a study of Indian states.

## Introduction

The Lives Saved Tool (LiST) (Walker *et al.*, 2013; LiST, 2017) is a publicly available tool that is designed to model the impact that scaling up key health interventions would have on outcomes of public health interest. The model maps changes in the coverage of specific interventions into changes in outputs such as wasting or stunting rates and birth outcomes. These are considered population risk factors for maternal and child mortality (Clermont and Walker, 2017; Mayberry and Morris, 2017). Widely used, the LiST has been employed, e.g., in projections of improvements in maternal and child mortality (Chou *et al.*, 2017) and for models of changes in child undernutrition (Bhutta *et al.*, 2013; Shekar *et al.*, 2017).

The underlying model hones-in on expected impacts of health interventions based on systematic reviews following standard criteria for evidence developed by the Child Health Epidemiology Reference Group (Walker *et al.*, 2010). For example, Bhutta *et al.* (2013) used the LiST to model impacts of health interventions and showed that if populations can access 10 evidence-based nutrition interventions at 90% coverage, under-5 mortality can be reduced by 15% and stunting burden can be averted by 20%. The evidence is evaluated for both quality and consistency, with randomized controlled trials (RCTs) given higher weight than observational studies, although adjustments are made in both categories; heterogeneity is also considered in the assessment of the data. Nevertheless, as is well established, the measures that ensure internal validity of evidence are not always adequate to guarantee external validity (Deaton and Cartwright, 2018). This is pertinent since many of the underlying meta-analyses for the LiST are based largely on relatively few efficacy trials, often with high supervision rates and comparatively small samples. The nature of these trials is relevant to the application of the LiST as is illustrated, e.g., in a study that compared development-related RCTs run by government agencies with those run by academics or by NGOs and found that the former generally had smaller effect sizes than the latter (Vivalt, 2016).

Studies that compare predictions by replicating the controlled conditions that generated the evidence are a first level of validation of the LiST. However, these need to be complemented by additional approaches that extrapolate beyond controlled studies. An example of previous comparisons of predictions from the LiST is an exercise that matched predictions generated by the LiST for four trials in South Asia with observed results; however, in those trials too, the delivery of the intervention package was assured and quality-controlled (Friberg *et al.*, 2010). It is plausible that interventions at scale will have different supervision ratios and other implementation features than RCTs; in such cases, the LiST may perform differently. Although many of the published applications of the LiST estimate the projected outcomes should different levels of coverage be achieved, few studies have assessed the extent to which the LiST is able to accurately estimate actual changes seen at scale in natural program implementation settings. Four studies, two in Niger (Amouzou *et al.*, 2012; Besada *et al.*, 2016), one in Tanzania (Afnan-Holmes *et al.*, 2015) and one in Malawi (Kanyuka *et al.*, 2016), have investigated how predicted progress in these outcomes over two periods compared to observed outcomes. All these studies projected under-five mortality rates higher than the observed; i.e., they underestimate the improvements. Other studies also used retrospective the LiST to examine whether changes in intervention coverage could account for neonatal mortality reduction (Khan *et al.*, 2012; Mbonye *et al.*, 2012; Pradhan *et al.*, 2012; Rubayet *et al.*, 2012; Zimba *et al.*, 2012). While prediction gaps were implicit in these studies, they were not a focus and stunting was included only as a driver.

We contribute to the limited global evidence on the performance of the LiST by using the model to project the expected impact of scaling up health programs in India between 2006 and 2016 and comparing projected estimates to observed outcomes in 2016. Our study is significant in that it is conducted over a period when India expanded programs to address reproductive, maternal, newborn health, child health and nutrition through the establishment of a massive new national health program and through expansion of a nutrition-focused program (Rao and Kaul, 2018; Chakrabarti *et al.*, 2019).

## Methods

### Data sources

Data on demographic characteristics, intervention coverage, and child stunting and mortality were obtained from nationally representative household surveys. The base year indicators were obtained from the third round of India’s National Family and Health Survey (NFHS-3), conducted in 2005–6 (IPPS, 2007). The follow-up indicators were obtained from the recently released NHFS-4 (conducted in 2015–16) (IIPS, 2017). India’s NFHS is similar to the Demographic and Health Surveys conducted in other countries. Both these surveys are based on a multi-stage cluster sample design, covering large sample sizes (109 041 households from NFHS-3 and 601 509 households from NFHS-4) and provide information on the health and nutrition of women and children in India. Base year population estimates are obtained from the Census of India 2011 (RGCC, 2011).

### Outcome

Child mortality rate was estimated based on births and infant and child deaths reported by women age 15–49 as of the interview date, with the reference period being the 5 years preceding the survey. Child undernutrition, including stunting, underweight and wasting, was estimated among all children under 5 years of age. We also estimated anaemia prevalence among pregnant and non-pregnant women of reproductive age. Definitions of outcome indicators used in the LiST analyses were presented in Supplementary Table S1.

### Intervention coverage

We identified several nutrition-specific interventions across the lifecycle, including interventions affecting pregnancy, birth and infancy as well as household drinking water and sanitation (Black *et al.*, 2013). Coverage indicators were available for the most recent birth in the 5 years preceding each survey. Three interventions during pregnancy were included in the LiST: tetanus toxoid vaccination, iron–folic acid (IFA) supplementation and food supplementation during pregnancy. Two key indicators covering births were included: skilled birth attendance and health facility delivery. A wide range of interventions during infancy are used in the LiST, including immunization, vitamin A supplementation, oral rehydration solution (ORS) and zinc provision during diarrhoea. In addition, the LiST includes infant and young child feeding (IYCF) practices as a proxy for effective programs to support optimal IYCF. Definitions of indicators of intervention coverage used in the modelling projections are provided in Supplementary Table S1.

### The LiST estimation

We used the Lived Saved Tool (LiST) version v5.63 to project the potential impact of changes in intervention coverage from 2006 to 2016 on child mortality based on measured base year values of child mortality in 2006. Some interventions have a relatively simple relation with mortality, but some have more complex linkages, with impacts on multiple causes of mortality and on various risk factors. All the effect sizes have been preloaded in the LiST visualizer which contained interactive links that provide extensive information on effect sizes and reference (The LiST Visualizer https://www.livessavedtool.org/resources). We also estimated the impact of changes in coverage of interventions on other outcomes including child stunting and wasting as well as anaemia among pregnant women and women of reproductive age. We then examined the extent to which the number of child deaths averted and the number of stunting cases prevented could be associated with changes in intervention coverage between 2006 and 2016. We present the estimates of lives saved in 2016 relative to 2006 rather than cumulative annual estimates for 2006–16 because estimates of annual intervention coverage between 2006 and 16 were not available and had to be interpolated.

In addition to national-level estimates, we also examined child mortality and stunting at the state level. We selected 10 states with the largest number of stunted children for this analysis. The absolute numbers of stunted children for each state were calculated by multiplying the stunting prevalence for that state in 2006 with the estimated number of children 0–5 years of age per state from the Census of India 2011 (Supplementary Table S2). These 10 states of India contained ∼59.3 million stunted children, accounting for 78% of stunted children and 79% of the under-five mortality in the country.

For the national projections, we used the preloaded LiST default database for India in the base year. We validated this data with the NFHS-3 dataset and, in a few cases, modified the values to update them. For subnational projections at the state level, we used the Subnational Wizard from the LiST to input available state-level data on population, total fertility rate, mortality rates for infant and children under 5 years, stunting and wasting distributions, breastfeeding practices and several intervention coverages from the same sources as the national data. For inputs that were not available at subnational areas [e.g. cause of death structure, birth outcomes (preterm and small for gestational age), micronutrient deficiencies, etc.], the Wizard uses the LiST to project the missing data based on the difference between national and subnational intervention coverage.

### Comparisons of the LiST-based estimates to observe survey-based changes (2006–16)

We compared the changes in mortality and other outcomes produced by the LiST with measured outcomes from NFHS-4. The prediction gap was defined as the difference between the predicted change and the actual observed levels in NFHS-4. This could be positive or negative. We also estimated the correlation between prediction gap and actual changes in under-five mortality rates at the state level. We applied both direct inputs of stunting and wasting (by entering data on stunting and wasting distribution) and indirect inputs (changes in stunting distribution calculate based on changes in coverage of interventions linked to stunting).

## Results

**Figure 1:**
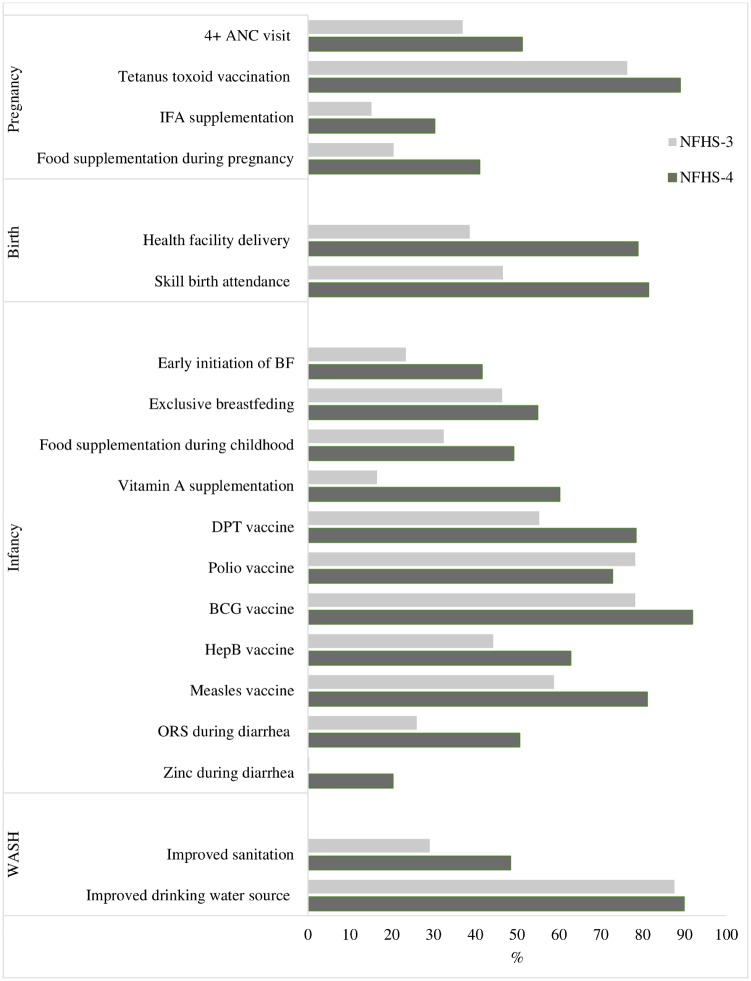
National coverage levels for high-impact interventions across the continuum of care in India between 2006 and 2016 utilized as inputs for the LiST.

The coverage of nutrition-specific interventions improved substantially between 2006 and 16 (Figure 1). The proportion of women who had at least four antenatal care visits during pregnancy increased from 37% to 51% and protection against neonatal tetanus improved from 76% to 89%. Consumption of IFA supplements during pregnancy doubled from 15% in 2006 to 30% in 2016. Similar changes were observed for food supplementation during pregnancy (20.5–41%). Deliveries at health facilities and births assisted by skilled birth attendants improved remarkably, reaching around 80% in 2016. For childhood interventions, early initiation of breastfeeding increased by 18 percentage points (pp), and supplementary feeding by 17 pp. Although exclusive breastfeeding increased by 9 pp (mainly due to reductions in the reported categories of predominant breastfeeding and of partial breastfeeding), non-breastfeeding also increased 3 pp for children 1–5.9 months. The coverage of immunization and vitamin A supplementation improved substantially in the last decade, and the proportion of children receiving ORS during diarrhoea also increased. Use of improved sanitation facilities improved, but even in 2016, <50% of households reported using such facilities.

**Figure 2:**
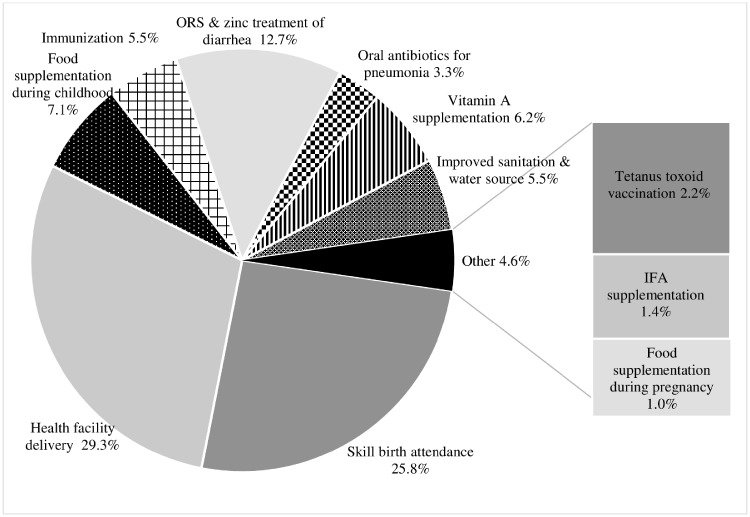
The LiST-based estimations of contributors to lives saved among children under 5 years of age in India between 2006 and 2016, by intervention (total under-five lives saved in 10 years 2006–16: 448 324. Although EBF increase, non-BF also increase, therefore, breastfeeding did not save lives, but increase death—20 512).

We estimated the number of child deaths that could be averted in India by changing the coverage of key nutrition-specific interventions and related health programs during pregnancy, birth and infancy from 2006 to 2016 levels, keeping defaults for other interventions. The LiST projects that the observed changes in coverage of included interventions between 2006 and 2016 would prevent an additional total of 448 324 deaths in children under 5 years from base year 2006 (272 652 lives saved among children <1 month and 175 672 lives saved among children 1–59 months) (Figure 2). The largest number of deaths predicted to be averted is due to changes in coverage of skilled birth attendance (26%). The two packages at birth (changes in health facility delivery and skilled birth attendance coverage) are estimated to contribute to 36% of the total lives of children younger than 5 years saved. Changes in coverage of postnatal care packages contributed to 19% lives saved. Scaling up IYCF interventions (complementary feeding promotion) contributed ∼7% of the lives saved in the projections. ORS and zinc treatment for diarrhoea together with vitamin A and zinc supplementation would save approximately 18% of the total. Similar results were observed for the direct entry method (Supplementary Table S3).

**Figure 3:**
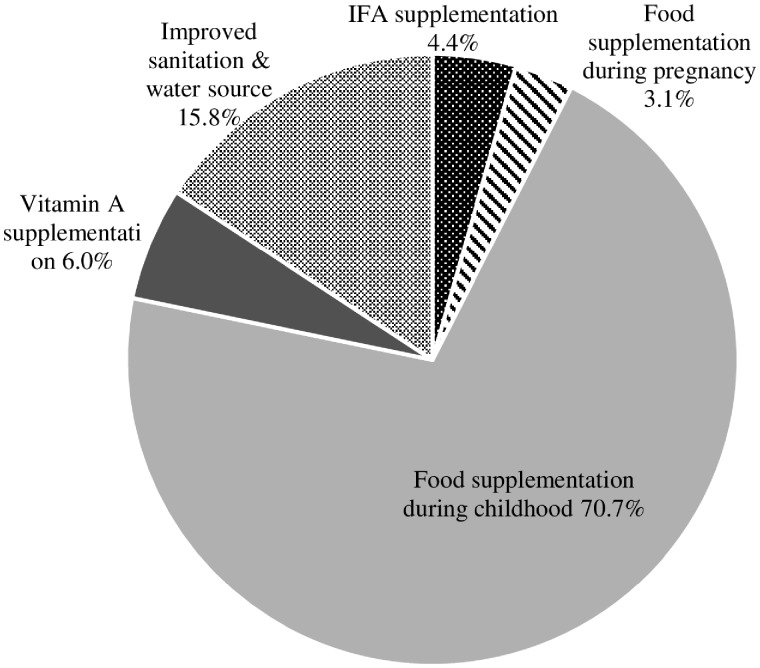
The LiST-based estimations of contributors to stunting cases prevented among children under 5 years of age in India between 2006 and 2016, by intervention (total stunting cases prevented in 10 years 2006–16: 4 590 908 children).

For stunting, the model estimated that 4.6 million cases of stunted children under 5 years of age would be averted by scaling up several interventions from 2006 to 2016. The key estimated contribution for stunting reduction came from expanded supplementary feeding (71%), followed by improved sanitation and water source (16%) and vitamin A supplementation (6%) (Figure 3).

At the national level, starting at a base year mortality rate in 2006 for children under 1 year of 57 per 1000 live births [95% confidence interval (CI) 52.4–61.6] and for children younger than 5 years of 74 per 1000 livebirths (95% CI 71.7–76.3), the LiST predicted mortality in 2016 at 42.6 (95% CI 40.8–44.4) and 56.4 (95% CI 55.5–57.3), respectively (Table 1). These estimations were very close to the observed values of mortality rate in 2016, at 41 (95% CI 39.2–42.8) and 50 (95% CI 49.2–50.8), respectively, per 1000 live births. Other estimations for stunting, wasting and anaemia at national level were also close to the actual values of NFHS-4, with small differences (between 2 pp and 5.6 pp). With the exception of wasting, the outcomes, as observed in NFHS-4, imply greater improvements than those predicted using the LiST.

**Table 1:** Comparison of LiST-based estimations of health and nutrition outcomes with observed outcomes in 2016: National Estimates

Outcomes	NFHS-3	NFHS-4	LiST Estimates	Prediction gap
	(Baseline)	(Endline)	(Projection 2016)	
Infant mortality rate	57.0 (52.4–61.6)	41.0 (39.2–42.8)	42.6 (40.8–44.4)	1.6
Under 5 mortality rate	74.0 (71.7–76.3)	50.0 (49.2–50.8)	56.4 (55.5–57.3)	6.3
Stunting among children under 5 years of age	48.0 (47.6–48.4)	38.4 (38.2–38.6)	44.0 (43.8–44.2)	5.6
Wasting among children under 5 years of age	19.8 (19.5–20.1)	21.0 (20.8–21.2)	19.1 (18.9–19.3)	−1.9
Anaemia among pregnant women	57.9 (56.2–59.7)	50.3 (49.6–51.2)	55.2 (55.0–55.4)	4.9
Anaemia among women of reproductive age	55.3 (54.5–55.9)	53.0 (52.9–53.5)	55.2 (55.0–55.4)	2.2

*Source of observed outcomes*: National Family Health Surveys for 2006 and 2016.

**Figure 4:**
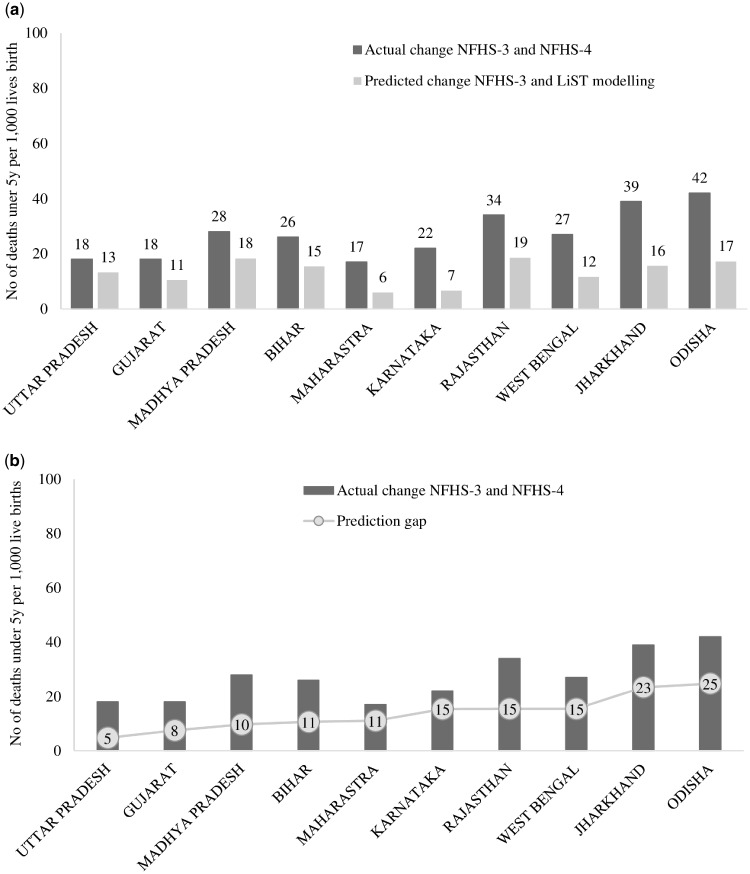
(a) Comparison of changes in under-five mortality predicted by the LiST with actual changes over the same period (India, 2006–16). (b) Relation between prediction gaps and actual changes in under-five mortality rates (India, 2006–16).

At the state level, we observed lower changes from the LiST projections of the under-five mortality rates compared to the actual changes between NFHS-3 and NFHS-4 for all states (Figure 4a), with the prediction gap ranging from 5 to 25 deaths per 1000 live births) (Figure 4b). We observed a correlation of 0.75 between prediction gap and the actual changes between NFHS-3 and NFHS-4, in which higher the actual change, the higher was the prediction gap (Figure 4b). The predicted changes using the LiST ranged from 30% of the actual value for Karnataka to 74% for Uttar Pradesh.

**Figure 5:**
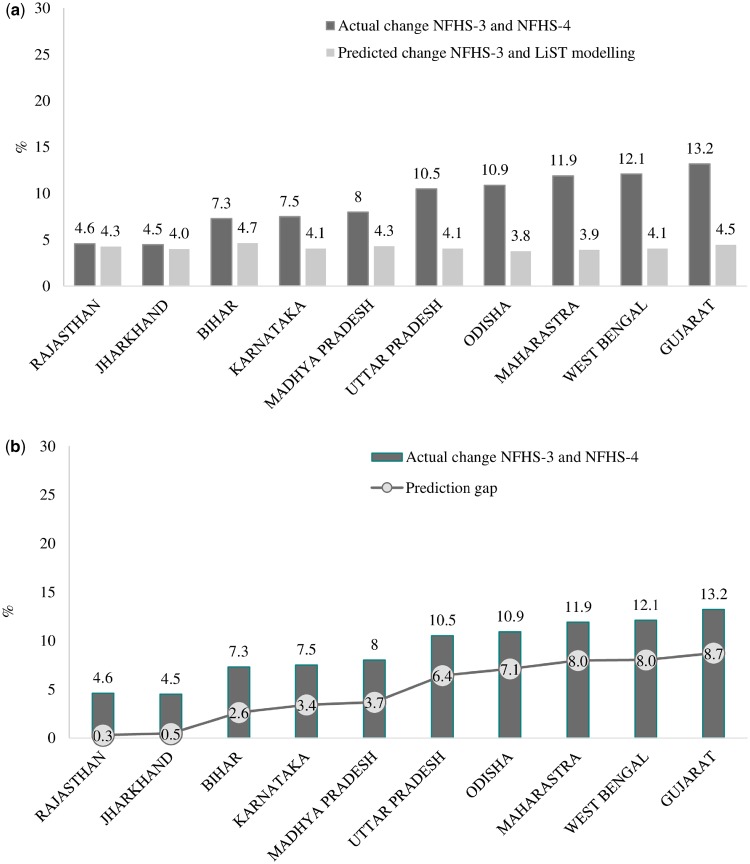
(a) Comparison of the LiST projections with the observed levels of stunting among children under 5 years of age in different states (India, 2006–16). (b) Relation between prediction gaps and actual changes in stunting (India, 2006–16).

For stunting, the actual changes ranged from 4.5–4.6 percentage points (pp) for Rajasthan and Jharkhand to 13.2 pp for Gujarat. However, the projected changes from the LiST were much less, ranging from only 3.9 pp to 4.5 pp (Figure 5a). The predicted changes using the LiST ranged from 34% of the actual value for Gujarat to 93% for Rajasthan (Figure 5b). As in the case of mortality, the higher actual change in stunting, the greater the difference between the LiST projection and actual value observed.

## Discussion

Policy planners use the LiST both to set feasible targets for mortality reduction and to prospectively determine which interventions are likely to contribute to realizing these goals and retrospectively to decompose drivers of observed changes. Similarly, the tool has been adapted to guide allocations of limited health budgets (Pearson *et al.*, 2018). The current study is intended to add to assessments of the degree to which one should have confidence that the scaling up from controlled studies to large populations and multi-year planning cycles provides plausible and realistic projections at the national and subnational level. Given that the LiST is continually being adapted, assessments of gaps in predications point to areas for potential improvement.

At the national level for India, the reductions in stunting and in child mortality that the LiST predicts based on changes in coverage of services between 2006 and 2016 are similar to—indeed slightly less than—the actual outcomes as observed in NFHS-4. Predictions of mortality improvements seem to be closer to the actual than those for improvements in stunting. The model also allows a decomposition of the results into shares associated with coverage of different interventions, e.g., it suggests that the increase in skilled birth attendance accounted for 26% of mortality decline. This cannot, however, be interpreted as a causal statement and indeed, is not fully in accord with other evidence for India (Lim *et al.*, 2010).

Some additional features of the model seemingly would lead to over- rather than underestimates of improvements. For example, the model automatically estimates coverage of delivery packages such as essential care, basic and comprehensive emergency obstetric services and newborn care when information on skilled birth attendance and health facility delivery are entered. The default assumption is that all basic care, either at home or at facilities provides clean birth practices, immediate assessment and stimulation of the newborn as well as labour and delivery management. Thus, there is an assumed relationship of service quality and attendance that may or may not prevail. In fact, findings from a study on quality using nationally representative data from 2012 to 2014 highlights that many Indian public primary care facilities fail to meet the nationally set minimum recommended standards for basic delivery and newborn care (Sharma *et al.*, 2018).

Similarly, in the model delivery in health facilities implies additional interventions such as neonatal resuscitation, antibiotics for premature rupture of the membranes, magnesium sulfate for management of eclampsia and active management of the third stage of labour. Likewise, comprehensive care assumed all the above components, with the addition of induction of labour for pregnancies lasting 41+ weeks. Logically, overestimating these services should lead to an overestimation of the impact of program availability. However, this is not apparent in the results. One likely offsetting factor may be the role of distal variables such as increases in per capita income or higher levels of maternal education. The LiST assumes that these changes only influence mortality by increasing the coverage of interventions or by reducing risk factors (Walker *et al.*, 2013). However, these distal variables also determine household purchases of food and other health inputs as well as the ability of a caregiver to avail of information provided by health professionals. Changes in these household characteristics may explain a fair portion of the positive prediction gap or even its entirety. The LiST also does not directly examine the role of ecological factors—such as environmental enteropathy—which interact with nutritional status. This is a limitation of the LiST and, by extension, the current study; as we do not determine the impact of service expansion per se holding income and education constant, we cannot indicate the performance of the tool in this application that health planners may often require.

All available data from NFHS that would impact outcomes were included in the current application of the LiST, but several coverages or interventions were not available in the NFHS data. These included folic acid supplementation/fortification before pregnancy, safe abortion services, calcium supplementation during pregnancy, case management for hypertension, diabetes and malaria during pregnancy; as well as full supportive care for neonatal sepsis/pneumonia during childhood. We cannot establish the degree to which these interventions improved over time, therefore, we cannot estimate their influence on the outcomes. This limitation may lead to underestimates of improvements.

The version of the LiST that was employed in this study updated earlier versions by allowing users to include evidence on supplementary feeding when modelling changes in intervention coverage on both stunting and wasting (Panjwani and Heidkamp, 2017). This update is based on evidence that complementary food supplementation interventions with or without nutrition education can have a small but significant effect in food-insecure settings on both linear and ponderal growth (Lassi *et al.*, 2013; Christian *et al.*, 2015). The evidence for India on the efficacy of complementary food supplements (Avula *et al.*, 2017) and the effectiveness of supplements delivered through the Integrated Child Development Services is, however, thin (Kandpal, 2011). Nevertheless, because supplementary feeding more than doubled between 2006 and 2016, the model indicates a large contribution from the increased coverage. In the estimates modelled here, we found 71% of child stunting was prevented by a combination of age-appropriate complementary feeding and the expansion of supplementary foods.

Improvements in anthropometric outcomes generally lead to improvements in mortality. In the LiST, one can enter changes in levels for stunting and wasting directly into projections to model mortality or have these rates influence mortality indirectly through changes in these outcomes that stem from the various interventions modelled. This article focused on the latter mechanism. However, using the former approach gives similar results (Supplementary Table S3). However, neither the direct nor the indirect modelling captures any possible interactions between wasting and stunting.

Maternal body mass index also has an impact on mortality, not through the direct impact of changes in body mass but as an indicator of food security. However, recent analysis concludes that there is no single indicator identified that is ideal for measuring the percentage of the population which is food secure (Jackson *et al.*, 2017). Moreover, while changes in food security have the potential to improve mortality as well as nutrition it is not an intervention per se but an outcome of other interventions and thus is a bit of a special case for the LiST as are IYCF practices which also are indicators of food security in the LiST.

The model performs well for predicting changes in mortality despite the lack of data on treatment of severe or moderate acute malnutrition which often contributes to a significant share of mortality reduction (Bhutta *et al.*, 2013). As data on coverage for interventions addressing severe acute malnutrition are unavailable for India, this is perhaps one area where future gains may be achieved with program scale-up. In contrast, the current study finds that health facility delivery and skilled birth attendance contributed to a large portion of under-five mortality reduction in the last 10 years. However, these have now reached close to 90%, leaving little potential for improvements in coverage in the future, rather than through quality improvements.

Estimated changes in child mortality at the national level are close to observed changes. However, changes from the LiST projection for state-level estimates of under-five mortality were smaller than the actual changes between NFHS-3 and NFHS-4 for all states. Possible explanations for this may be that for the national level, the LiST has a more comprehensive list of input data, either from previous datasets from India, or from other information on the aetiology of child deaths, common pathogens, the age distribution of fertility, sex ratio at birth and so on. For the subnational estimates, although the NFHS-4 data are available for total fertility rate, breastfeeding practices and coverage of several interventions, there are gaps in available data on other inputs (mentioned above). For inputs that were not available at subnational areas (e.g. cause of death structure, birth outcomes such as preterm and small for gestational age, micronutrient deficiencies and others), the LiST projects the missing data based on the difference between national and subnational intervention coverage.

The near saturation of coverage in some states may also be a partial explanation for anomalies in state-level results. For example, states like Tamil Nadu and Kerala had high rates of exclusive breastfeeding in 2006, thus offering limited scope for improvement. Thus, in these states there was no contribution of this important aspect of childcare to improved mortality. Indeed, in Kerala there was a small increase in mothers that reported no breastfeeding. Since the LiST uses exclusive breastfeeding as one element of its calculations, this drove projections of a slight decline in mortality for children 1–59 months that offset other improvements in neonatal mortality, which given the relatively low rates at baseline for this state were comparatively modest.

## Conclusion

Many countries—as well as subnational entities like states in India—are setting targets for improvements in mortality and in anthropometric indicators. Knowing how much the expansion of service coverage can potentially support the achievement of such goals can be useful to policymakers and technical stakeholders. While the LiST does not replace the need for program impact evaluation it can help in estimating potential impacts, at full scale in a timely manner. This work, examining projected trends vs actual outcomes over a decade in India, the country with the largest number of child deaths and stunted and anaemic children in the world, sheds light on the tool performance at the national and subnational level. It both provides some assurance that existing applications of the LiST are informative as well as highlights some of the challenges that need to be addressed to further strengthen the use of modelling tools such as the LiST for priority-setting in public health.

*Ethical approval.* No ethical approval was required for this study.

## Supplementary Material

czz088_Supplementary_Data
