# Supplementary material for: Progress in reducing child mortality and stunting in India: an application of the Lives Saved Tool
Source: Health Policy Plan. 2019 Sep 16;34(9):667–75. doi: 10.1093/heapol/czz088 (PMC6880331; doi:10.1093/heapol/czz088)
Supplement: czz088_Supplementary_Data [file czz088_supplementary_data.zip › czz088-Suppl_data/Supplementary_Table3.doc]

**Supplementary Table 3: Comparison of findings from the direct and indirect entry of stunting and wasting in the LiST to estimate lives saved among children under 5 years of age, by intervention**

| **Interventions** | **Direct entry method** | | **Indirect entry method** | |
| --- | --- | --- | --- | --- |
|  | **No of lives saved** | **% share** | **No of lives saved** | **% share** |
| Tetanus toxoid vaccination | 9,811 | 2.2 | 9,811 | 2.2 |
| IFA supplementation | 5,058 | 1.1 | 6,306 | 1.4 |
| Balanced energy supplementation | 3,534 | 0.8 | 4,406 | 1.0 |
| Skilled birth attendance | 115,620 | 25.4 | 115,620 | 25.8 |
| Health facility delivery | 131,258 | 28.8 | 131,258 | 29.3 |
| Supplementary feeding | -- | 0.0 | 31,767 | 7.1 |
| Change in stunting prevalence | 59,561 | 13.1 | -- | -- |
| Immunization | 24,546 | 5.4 | 24,677 | 5.5 |
| ORS and zinc treatment of diarrhea | 56,138 | 12.3 | 56,983 | 12.7 |
| Oral antibiotics for pneumonia | 14,613 | 3.2 | 15,006 | 3.3 |
| Vitamin A supplementation | 26,184 | 5.7 | 27,765 | 6.2 |
| Improved sanitation and water source | 22,733 | 5.0 | 24,725 | 5.5 |
| Change in wasting prevalence | -13,596 | -3.0 | -- | -- |
| Total lives saved | 455,460 | 100.0 | 448,324 | 100 |
