# Supplementary material for: Progress in reducing child mortality and stunting in India: an application of the Lives Saved Tool
Source: Health Policy Plan. 2019 Sep 16;34(9):667–75. doi: 10.1093/heapol/czz088 (PMC6880331; doi:10.1093/heapol/czz088)
Supplement: czz088_Supplementary_Data [file czz088_supplementary_data.zip › czz088-Suppl_data/Supplementary_Table1.doc]

**Supplementary Table 1: Definition of outcome and coverage indicators used in the** LiST analyses

| A. **INDICATORS** | **DEFINITION** |
| --- | --- |
| **Outcomes** | |
| Infant mortality rate | The number of deaths of children under one year of age per 1000 live births born in the five years preceding the survey. |
| Under five mortality rate | The number of deaths of children under five years of age per 1000 live births born in the five years preceding the survey. |
| Stunting among children under five years of age | Percentage of children 0–59 months who are <-2SD from median height for age of the WHO Child Growth Standards |
| Wasting among children under five years of age | Percentage of children 0–59 months who are below <-2SD from median weight for height of the WHO Child Growth Standards |
| Anemia among women of reproductive age | Percentage of women 15-49 years old who are anemic (<12.0 g/dl for non-pregnant women and <11.0 g/dl for pregnant women) |
| Anemia among pregnant women | Percentage of pregnant women who are anemic (<11.0 g/dl) |
| **Coverage indicators** |  |
| **Pregnancy** |  |
| ANC (four or more visits) | Percentage of mothers receiving at least four ANCs for the last birth in the last five years |
| ANC-neonatal tetanus injections | Percentage of mothers who were protected against neonatal tetanus for the last birth in the last five years |
| Consumption of IFA supplements | Percentage of mothers who took IFA supplements for at least 100 days for the last birth in the last five years |
| Food supplementation (pregnancy) | Percentage of mothers with children under age six years in areas covered by an AWC who received supplementary nutrition from the AWC during pregnancy |
| **Birth** |  |
| Skilled attendant at birth | Percentage of births in the last five years attended by skilled health personnel |
| Delivery in a health facility | Percentage of births delivered in a health facility for births in the last five years |
| **Infancy** |  |
| Early initiation of breastfeeding | Percentage of children 0-23.9 months who were breastfed within one hour of birth |
| Exclusive breastfeeding | Percentage of children 0-6 months of age who are fed exclusively with breast milk |
| Supplementary feeding | Percentage of children 6–35 months covered by AWCs who received supplementary food provided at the AWC in the last 12 months |
| BCG vaccine | Percentage of children aged 12‐23 months who received Baccille Calmette Guérin vaccine |
| Polio vaccine | Percentage of infants who received vaccine to prevent Poliomyelitis disease |
| DPT vaccine | Percentage of infants who received three doses of diphtheria–tetanus–pertussis vaccine |
| HepB vaccine | Percentage of infants who received vaccine to prevent chronic Hepatitis B virus (HBV) infection |
| Measles vaccine | Percentage of infants who received the first dose of measles vaccine |
| Vitamin A | Percentage of children age 9–59 months who received vitamin A supplements |
| ORS during diarrhea | Percentage of children under five years with diarrhea in the last 2 weeks who received ORS |
| Zinc during diarrhea | Percentage of children under five years with diarrhea in the last 2 weeks who received Zinc |
| **Wash** |  |
| Improved sanitation | Percent distribution of households using improved sanitation facilities |
| Improved drinking water | Percent distribution of households with an improved drinking water source |

| B. **DEFAULT INTERVENTIONS** | **Value used (%)** |
| --- | --- |
| ***Preconceptual*** |  |
| Folic acid supplementation/ fortification | 0.0 - unchanged |
| Safe abortion services | 35.1 - unchanged |
| ***Pregnancy*** |  |
| Syphilis detection and treatment | 18.2 - unchanged |
| Calcium supplementation | 0.0 - unchanged |
| Hypertensive disorder case management | 45.2 - unchanged |
| Diabetes case management | 15.7 - unchanged |
| Malaria case management | 51.8 - unchanged |
| MgSO4 management of pre-eclampsia | 6.9 - unchanged |
| Fetal growth restriction detection and management | 0.0 - unchanged |
| ***Childhood*** |  |
| Case management of premature babies | 38.7🡪 78.9 |
| Case management of neonatal sepsis/pneumonia | 38.7🡪 78.9 |
| Antibiotics for treatment of dysentery | 15.5 - unchanged |
| Oral antibiotics for pneumonia | 69.1🡪 73.2 |
| ***Vitamin A for treatment of measles | 16.5🡪 60.2 |
| ACTs - Artemesinin compounds for treatment of malaria | 0.0 - unchanged |
| SAM - treatment for severe acute malnutrition | 0.0 - unchanged |
