# Supplementary material for: Progress in reducing child mortality and stunting in India: an application of the Lives Saved Tool
Source: Health Policy Plan. 2019 Sep 16;34(9):667–75. doi: 10.1093/heapol/czz088 (PMC6880331; doi:10.1093/heapol/czz088)
Supplement: czz088_Supplementary_Data [file czz088_supplementary_data.zip › czz088-Suppl_data/Supplementary_Table2.doc]

**Supplementary Table 2: Stunting and under-five mortality in ten large states within India that are included in the LiST subnational projections**

| **State** | **Stunting among children under 5y of age in 2016**  **(%)** | **Stunting among children under 5y of age in 2006**  **(%)** | **Population of children 0-6y of age (Census of India, 2011)** | **Total number of stunted children 0-5y of age (2006)** | **Under-5 mortality rate in 2006** | **Under-5 mortality rate in 2016** |
| --- | --- | --- | --- | --- | --- | --- |
| **INDIA** | **38.4** | **48.0** | **158,789,287** | **76,218,858** | **74** | **50** |
| *UTTAR PRADESH^1^* | *46.3* | *56.8* | *29,728,235* | *16,885,637* | *96* | *78* |
| *BIHAR* | *48.3* | *55.6* | *18,582,229* | *10,331,719* | *84* | *58* |
| *MAHARASTRA* | *34.4* | *46.3* | *12,848,375* | *5,948,798* | *46* | *29* |
| *MADHYA PRADESH* | *42* | *50.0* | *10,548,295* | *5,274,148* | *93* | *65* |
| *RAJASTHAN* | *39.1* | *43.7* | *10,504,916* | *4,590,648* | *85* | *51* |
| *WEST BENGAL* | *32.5* | *44.6* | *10,112,599* | *4,510,219* | *59* | *32* |
| *GUJARAT* | *38.5* | *51.7* | *7,564,464* | *3,910,828* | *61* | *43* |
| ANDHRA PRADESH | 31.4 | 42.7 | 8,642,686 | 3,690,427 | -- | 41 |
| *KARNATAKA* | *36.2* | *43.7* | *6,855,801* | *2,995,985* | *54* | *32* |
| *JHARKHAND* | *45.3* | *49.8* | *5,237,582* | *2,608,316* | *93* | *54* |
| *ODISHA* | *34.1* | *45.0* | *5,035,650* | *2,266,043* | *91* | *49* |
| TAMIL NADU | 27.1 | 30.9 | 6,894,821 | 2,130,500 | 35 | 32 |
| ASSAM | 36.4 | 46.5 | 4,511,307 | 2,097,758 | 84 | 56 |
| CHATTISGARH | 37.6 | 52.9 | 3,584,028 | 1,895,951 | 90 | 64 |
| HARYANA | 34 | 45.7 | 3,297,724 | 1,507,060 | 52 | 41 |
| PUNJAB | 25.7 | 36.7 | 2,941,570 | 1,079,556 | 52 | 33 |
| DELHI | 32.3 | 42.2 | 1,970,510 | 831,555 | 47 | 47 |
| KERALA | 19.7 | 24.5 | 3,322,247 | 813,951 | 16 | 7 |
| JAMMU & KASHMIR | 27.4 | 35.0 | 2,008,670 | 703,035 | 51 | 38 |
| UTTARAKHAND | 33.5 | 44.4 | 1,328,844 | 590,007 | 56 | 47 |
| MEGHALAYA | 43.8 | 55.1 | 555,822 | 306,258 | 70 | 40 |
| HIMACHAL PRADESH | 26.3 | 38.6 | 763,864 | 294,852 | 42 | 38 |
| TRIPURA | 24.3 | 35.7 | 444,055 | 158,528 | 59 | 33 |
| MANIPUR | 28.9 | 35.6 | 353,237 | 125,752 | 42 | 26 |
| NAGALAND | 28.6 | 38.8 | 285,981 | 110,961 | 65 | 37 |
| ARUNACHAL PRADESH | 29.4 | 43.3 | 202,759 | 87,795 | 88 | 33 |
| MIZORAM | 28.0 | 39.8 | 165,536 | 65,883 | 53 | 46 |
| GOA | 20.1 | 25.6 | 139,495 | 35,711 | 20 | 13 |
| SIKKIM | 29.6 | 38.3 | 61,077 | 23,392 | 40 | 32 |

^1^*Italics* are ten states with the largest number of stunted children that were selected for subnational analysis
